# A Palliative Care Approach to Amyotrophic Lateral Sclerosis

**DOI:** 10.7759/cureus.51048

**Published:** 2023-12-24

**Authors:** Cláudio Gouveia, Licínia Araújo, Susete Freitas, João Correia, Vilma Passos, Graciela Camacho, Luísa Gomes, Helena Fragoeiro, Cristiana Camacho, Beatriz Chambino

**Affiliations:** 1 Internal Medicine, Centro Hospitalar Lisboa Ocidental, Lisbon, PRT; 2 Palliative Medicine, Serviço de Saúde da Região Autónoma da Madeira (SESARAM), Funchal, PRT; 3 Internal Medicine, Hospital Central do Funchal, Funchal, PRT

**Keywords:** non-cancer palliative care, neurology, end-of-life, palliative medicine, amyotrophic lateral sclerosis

## Abstract

Introduction: Amyotrophic lateral sclerosis (ALS) is a degenerative disease characterized by motor dysfunction. Currently, treatment options are limited and management is based mostly on symptom control and quality of life optimization, so palliative care plays a fundamental role. Our objective was to characterize the ALS population in Madeira Island that was referenced and/or followed by a palliative care unit over a five-year period.

Methods: Longitudinal, retrospective, descriptive, and observational study to analyze patients with ALS who were referred and/or followed by a palliative care unit during a five-year period, between 2017 and 2021. Patient’s medical electronic and physical records were analyzed to gather data. Descriptive and inferential statistical analysis was done using Microsoft Excel and Statistical Package for the Social Sciences (version 28.0.1).

Results: During this five-year period, a total of 38 patients were diagnosed with ALS in Madeira Island and 23 (60.53%) were referred to palliative care. Three patients died before assessment, so 20 (50.63%) were followed by the palliative care team. They had a median life expectancy of 425 days and the median time spent in palliative care was 137 days. Of this population, 56.52% (n=13) was male with an average age of 64 years. The median period from diagnosis to referral was 167 days, with most referrals being made by family medicine (39.13%; n=9) motivated by uncontrolled symptoms (95.65%; n=22). The median period from referral to first assessment by a palliative care physician was 19 days. The Palliative Performance Scale (PPS) and Confusion Assessment Method (CAM) applied on the first visit had a median score of 40% in the former and was negative in 95.00% (n=19) of patients in the latter. Advanced care directives were present in 55.00% (n=11) of patients and all provided care was in accordance with the patient’s wishes. The most common symptoms were dysphagia, dyspnea, pain, anxiety, and sialorrhea. The most used drugs were morphine, riluzole, butylscopolamine, bisacodyl, and midazolam. As for other interventions, 55.00% (n=11) of patients underwent noninvasive ventilation (NIV), 15.00% (n=3) were submitted to percutaneous endoscopic gastrostomy (PEG), and one patient (5.00%) was nasogastrically intubated. The death rate was 95.00% (n=19) with 73.68% (n=14) of deaths occurring in the palliative care unit.

Conclusion: Literature has shown that there are many advantages to the early inclusion of palliative care in ALS management, achieving effective symptom control and greater quality of life, but also reducing caregiver burden. However, in this study, we found that referrals to palliative care were late and included mostly cases of advanced disease with uncontrolled symptoms.

## Introduction

Amyotrophic lateral sclerosis (ALS) is a neurodegenerative disorder affecting primarily the motor system, but extra-motor manifestations can also be present. ALS often has a focal onset but subsequently spreads to different body regions [1]. It causes weakness, muscle atrophy, fasciculations, and spasticity, which interfere with basic functions such as mobility, swallowing, and breathing; however, cognitive and behavioral alterations can also be present [2]. With disease evolution, the progressive loss of muscle strength leads to respiratory failure, with this being the most common cause of death in these patients [3].

The estimated incidence is 1.75-3 per 100000 people per year and a prevalence of 10-12 per 100000 people in Europe [1]. As for etiology, in 10% of patients, family history suggests an autosomal dominant inheritance pattern; however, the remaining 90% have no affected family members and are classified as sporadic forms of the disease. Causes seem to be heterogeneous and are only partially understood [4].

Treatment options continue to be very limited, and clinical management mostly aims to control symptoms. The average survival in ALS is two to four years; nevertheless, some patients have a more slowly progressive disease form and can survive for over a decade, emphasizing the importance of symptomatic management and optimal quality of life throughout the course of the disease [5]. To this end, coordinated multidisciplinary care, which includes palliative medicine, is fundamental [3].

Due to the complex management of advanced ALS, guidelines recommend that specialist palliative care should be involved early and throughout the disease, thus making more time for the patient to participate in advanced care planning, which enables them to discuss and be involved in their own plan of care [5]. Palliative care is essential to maximize the quality of life of patients and families by relieving symptoms; providing emotional, psychological, and spiritual support; removing obstacles to a peaceful death; and supporting the family in bereavement [6]. 

## Materials and methods

The primary objective of this study was to characterize the ALS population in Madeira Island that was referenced and/or followed by a palliative care unit. This is the only palliative care unit on the whole island and is responsible for providing care to a total of around 250000 inhabitants.

Before starting data collection, a project proposal was drafted and submitted for evaluation by the hospital's ethics committee. After careful analysis, approval was acquired (IRB approval number: S.22002959) and the project could be started.

To characterize the ALS population, we wanted to look at different variables, such as gender, age, time from diagnosis to palliative care referral, referral motive, referring specialty, and time from referral to first assessment by a palliative care physician. We also wanted to analyze the Palliative Performance Scale (PPS) score at the first consult, the presence of delirium based on the application of the Confusion Assessment Method (CAM), the existence of advanced care directives regarding percutaneous endoscopic gastrostomy (PEG), nasogastric intubation, orotracheal intubation, tracheotomy, and advanced life support and if the provided care was in accordance. Finally, we also aimed to explore the most prevalent symptoms, most common treatments/drugs used, use of noninvasive ventilation (NIV), death rate, place of death, duration of palliative care, and life expectancy since diagnosis.

With this aim, a longitudinal, retrospective, descriptive, and observational study was developed to analyze patients with ALS, in Madeira Island, who were referred and/or followed by a palliative care team during a five-year period (from the 1st of January 2017 to the 31st of December 2021). Hospital records were consulted to determine the number of patients diagnosed with ALS during this period. Their medical electronic and physical records were analyzed to gather the previously cited data. Descriptive and inferential statistical analysis was done using Microsoft Excel and Statistical Package for the Social Sciences (SPSS) version 28.0.1.

## Results

**Figure 1:**
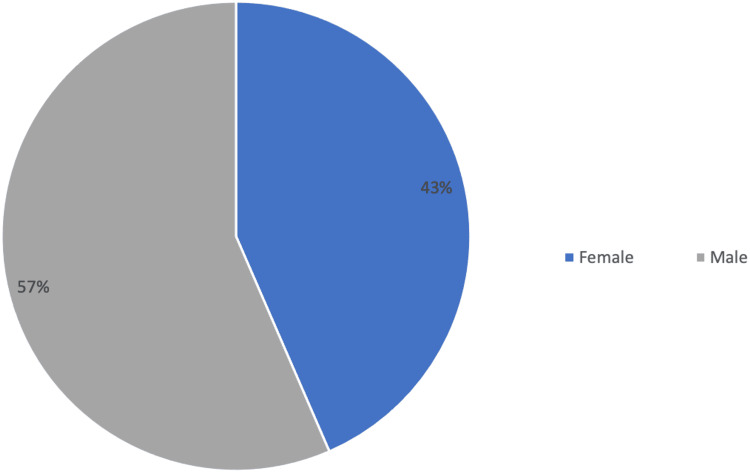
Patient distribution by sex.

**Figure 2:**
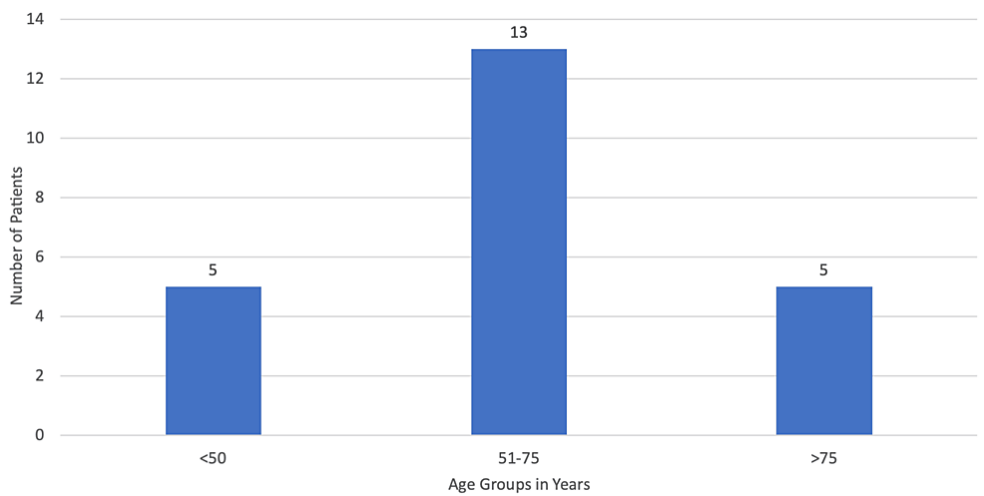
Patient distribution by age.

During this five-year period, a total of 38 patients were diagnosed with ALS in Madeira Island, with an estimated yearly incidence that varied from 2.4 to 3.6 per 100000 people. Of these patients, 23 (60.53%) were referred to palliative care. Three patients died before being assessed by a palliative care physician and consequently, only 20 patients (50.63%) were followed by the palliative care team. Of the 23 patients, 56.52% (n=13) were male and 43.48% (n=10) were female, with the average age being 64.3 years (minimum: 40 years; maximum: 85 years) as can be seen in Figure 1 and Figure 2.

**Figure 3:**
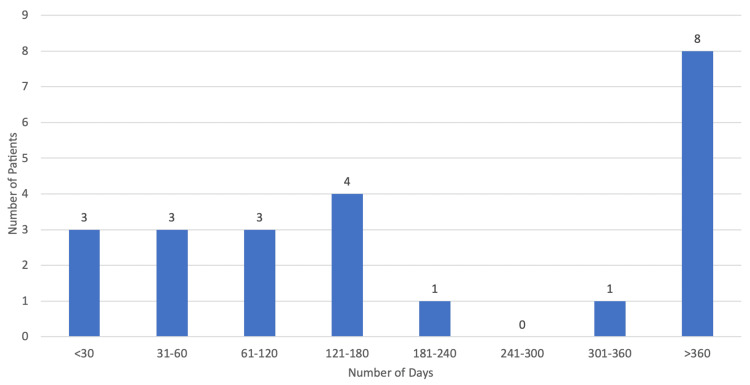
Period duration from diagnosis to palliative care referral.

**Figure 4:**
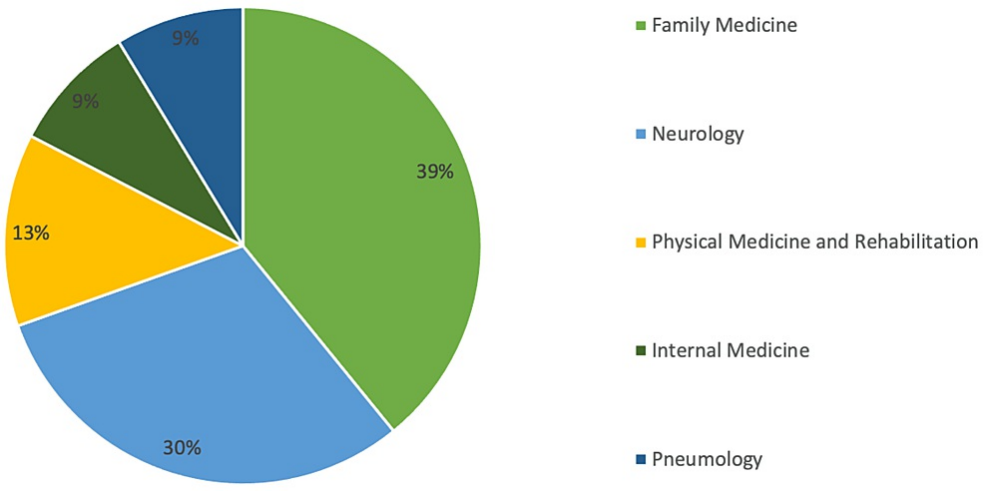
Referring specialties.

The median period duration from diagnosis to palliative care referral was 167 days (approximately six months) with the minimum duration being 16 days and the maximum being 712 days, which is approximately 24 months (Figure 3). As for the referral motive, 95.65% (n=22) of cases were due to decompensated symptoms with only one patient (4.35%) being referred at the time of diagnosis. When it came to the referring specialty (Figure 4), the majority of referrals came from family doctors (39.13%; n=9), followed by neurology (30.43%; n=7), physical medicine and rehabilitation (13.04%; n=3), internal medicine (8.70%; n=2), and pneumology (8.70%; n=2).

**Figure 5:**
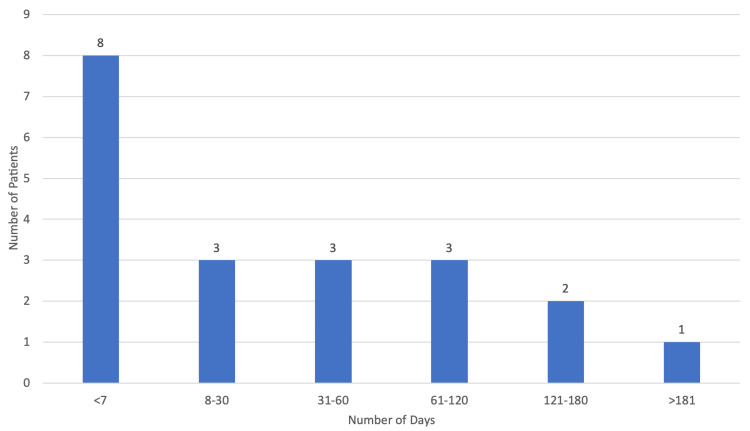
Period duration from referral to first evaluation by a palliative care physician.

**Figure 6:**
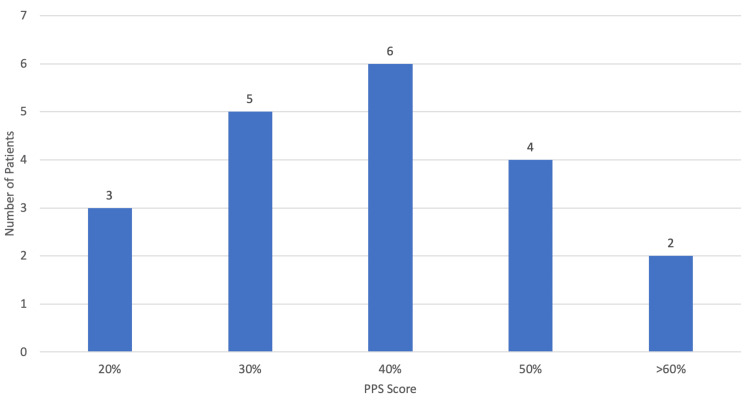
PPS score at first evaluation. PPS, Palliative Performance Scale

The median period duration from referral to first assessment by a palliative care physician (Figure 5) was 19 days, with the minimum waiting-time being zero days and the maximum being 187 days (approximately six months). At the first assessment, the median PPS was 40%, with the highest score being 70% and the lowest being 20% (Figure 6). Through the application of the CAM, it was determined that in most cases (95.00%; n=19) there was no delirium present, being positive in only one patient (5.00%).

Regarding advanced care directives, these were present in 55.00% (n=11) of patients, ten patients refused PEG, and four patients refused nasogastric intubation. Orotracheal intubation, tracheotomy, and advanced life support were refused by one patient each. All provided care was in accordance with the patient’s advanced directives.

A total of three patients (15.00%) underwent PEG, with one patient surviving 264 days (approximately nine months); however, the other two patients survived 92 and 93 days, respectively (approximately three months). Only one patient (5.00%) was submitted to nasogastric intubation and this was done 25 days before death occurred.

When it came to the most prevalent symptoms during the course of disease follow-up, these were dysphagia, present in 80.00% (n=16) of patients, followed by dyspnea (65.00%; n=13), pain (55.00%; n=11), anxiety (45.00%; n=9), sialorrhea (45.00%; n=9), insomnia (35.00%; n=7), constipation (35.00%; n=7), and muscle weakness (25.00%; n=5). In Table 1, there is a list of all the recorded symptoms described by the patients and the patient’s family and/or carers.

**Table 1:** List of symptoms described by patients, their families, and/or carers.

Symptoms	Number of patients (%)
Dysphagia	16 (80.00%)
Dyspnea	13 (65.00%)
Pain	11 (55.00%)
Anxiety	9 (45.00%)
Sialorrhea	9 (45.00%)
Insomnia	7 (35.00%)
Constipation	7 (35.00%)
Muscle weakness	5 (25.00%)
Emotional distress	3 (15.00%)
Cough	3 (15.00%)
Spasticity	2 (10.00%)
Myoclonus	2 (10.00%)
Cephalalgia	2 (10.00%)
Dysarthria	2 (10.00%)
Anorexia	2 (10.00%)
General asthenia	2 (10.00%)
Xerostomia	2 (10.00%)
Dysphonia	1 (5.00%)
Bruxism	1 (5.00%)
Delirium	1 (5.00%)
Nausea	1 (5.00%)
Drowsiness	1 (5.00%)
Tetraparesis	1 (5.00%)

As for the most frequent treatments/drugs used, it was observed that morphine was the most common drug present in the therapeutic plan, it was taken by 18 patients (90.00%). It was followed by riluzole (65.00%; n=13), butylscopolamine (40.00%; n=8), bisacodyl (35.00%; n=7), midazolam (35.00%; n=7), levomepromazine (30.00%; n=6), oxygen (30.00%; n=6), baclofen (30.00%; n=6), lactulose (25.00%; n=5), and paracetamol (25.00%; n=5). In Table 2, there is a list of all the recorded drugs taken by the patients. Concerning the use of NIV, the records showed that 55.00% (n=11) of patients underwent this treatment whereas 45.00% (n=9) did not.

**Table 2:** List of all the recorded drugs taken by the patients.

Drugs	Number of patients (%)
Morphine	18 (90.00%)
Riluzole	13 (65.00%)
Butylscopolamine	8 (40.00%)
Bisacodyl	7 (35.00%)
Midazolam	7 (35.00%)
Levomepromazine	6 (30.00%)
Oxygen	6 (30.00%)
Baclofen	6 (30.00%)
Lactulose	5 (25.00%)
Paracetamol	5 (25.00%)
Furosemide	4 (20.00%)
Salbutamol	3 (15.00%)
Atropine	3 (15.00%)
Macrogol + sodium chloride + sodium bicarbonate + potassium chloride	3 (15.00%)
Metamizole magnesium	3 (15.00%)
Lorazepam	3 (15.00%)
Alprazolam	3 (15.00%)
Fentanyl	3 (15.00%)
Metoclopramide	3 (15.00%)
Clonazepam	2 (10.00%)
Sertraline	2 (10.00%)
Ipratropium bromide	2 (10.00%)
Dexamethasone	2 (10.00%)
Senna	1 (5.00%)
Diazepam	1 (5.00%)
Olanzapine	1 (5.00%)
Quetiapine	1 (5.00%)
Haloperidol	1 (5.00%)
Gabapentin	1 (5.00%)
Tramadol	1 (5.00%)
Domperidone	1 (5.00%)
Sucralfate	1 (5.00%)
Paroxetine	1 (5.00%)
Mirtazapine	1 (5.00%)
Trazodone	1 (5.00%)

**Figure 7:**
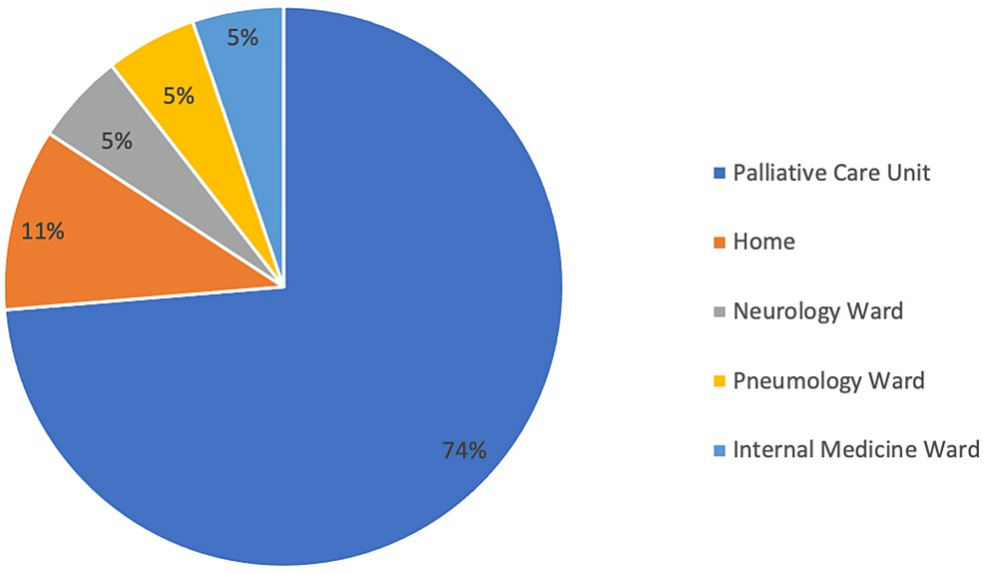
Patient's place of death.

**Figure 8:**
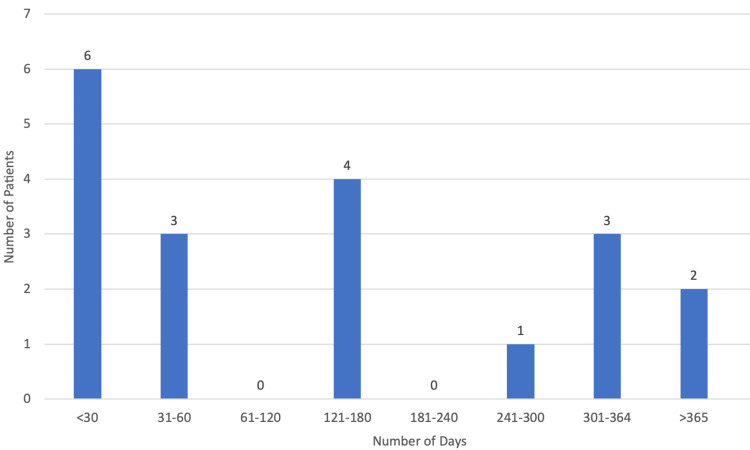
Duration of palliative care.

A total of 19 patients (95.00%) died during this five-year period, with only one patient (5.00%) surviving. As for the place of death (Figure 7), 14 patients (73.68%) died in the palliative care unit, with 10.53% (n=2) of patients dying at home, one patient (5.26%) in the neurology ward, another (5.26%) in the pneumology ward, and one last patient (5.26%) dying in the internal medicine ward. The median duration of palliative care (Figure 8) was 137 days (approximately five months) with the minimum being one day and the maximum being 474 days (approximately 16 months).

**Figure 9:**
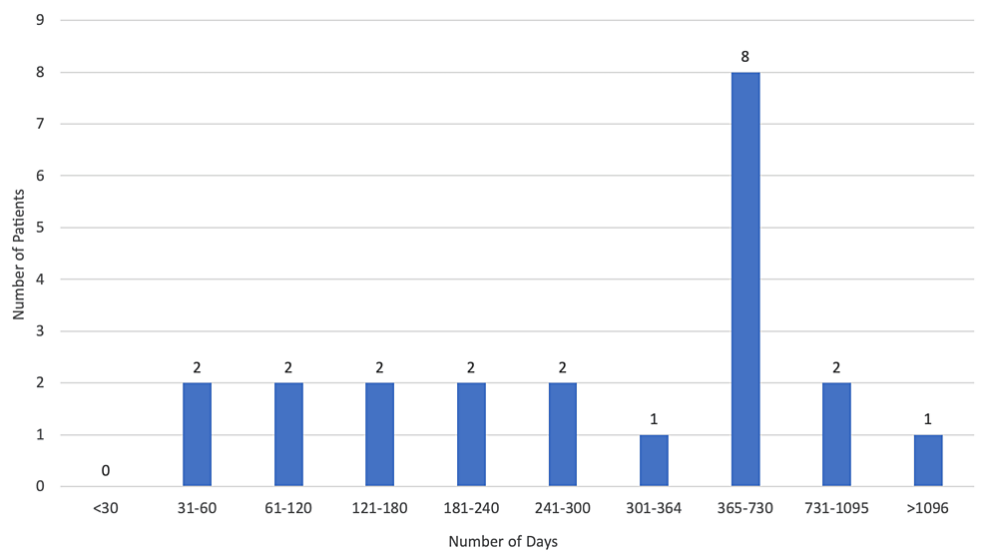
Patient's life expectancy since diagnosis.

The median life expectancy since diagnosis (Figure 9) was 425 days (approximately 14 months). As for the longest survival period, this was 1211 days (approximately 40 months) and the shortest was 39 days.

## Discussion

ALS is a relatively rare disease, with a cumulative lifetime risk of 1:350 in men and 1:400 in women, with an estimated incidence of 1.75-3 per 100000 people per year. The mean age for symptom onset can vary; when it comes to sporadic ALS, it can be 58-63 years but for familial ALS, it is 40-60 years [1]. During this five-year period, there was a total of 38 diagnosed patients. This is an incidence of 2.4 to 3.6 per 100000 people, which is in accordance with European data with the majority of this study population being male (56.52%; n=13) with an average age of 64.3 years.

Only 60.53% of patients (n=23) were referred for palliative care, which we suppose is mostly due to a lack of knowledge on the importance of palliative care in these patients. There is considerable evidence to suggest that the involvement of palliative care in a multidisciplinary approach to ALS helps to improve quality of life, symptom control, and caregiver burden [2]. The European Federation of Neurological Sciences’ task force recommends that palliative care should be involved from the time of diagnosis, with early referral being of the utmost importance, as sufficient time is essential to establish relationships with the clinical team and to address end-of-life matters [4]. This is particularly important as speech and communication are often limited in the later stages of disease [2]. However, in this study, the median period duration from diagnosis to palliative care referral was 167 days (approximately six months), with most referrals being made by family physicians and not neurologists and with pneumology being the least referring specialty. The majority of patients (95.65%; n=22) were referred due to decompensated symptoms with a median PPS score of 40%, suggesting advanced disease with significant functional decline. Only one patient (4.35%) was referred at the time of diagnosis. The median period duration from referral to first assessment by a palliative care physician was 19 days, and this slight delay is mostly explained by limited human resources. Nevertheless, these findings are actually quite common as there is currently no widely accepted international standard for the initiation of a palliative care intervention. What has been found is that management of terminal phases of ALS has been reported to be unsatisfactory in a high percentage of cases, with only a small fraction of patients having palliative care involvement from the moment of diagnosis [7].

The discussion of advanced care directives generally covers preferences for symptom management, resuscitation, and nutritional and respiratory support, including nasogastric intubation, gastrostomy insertion, tracheotomy, and noninvasive and invasive mechanical ventilation [3]. In this study, advanced directives were present in only half of patients, which can mostly be explained by the late referral of these patients for palliative care, usually with advanced disease and severe functional decline. This greatly limits the time and conditions to create a therapeutic relationship of trust with the patient and family, in order to have these discussions and make a plan of care. Research has suggested that patients with ALS generally welcome the opportunity to discuss end-of-life issues with their physician [8]. For this to happen, these patients should be approached early during the disease course by professionals with specific training with time to allow for reflection and integration of choices within the patient’s priorities and life plans. Most of the patients with advanced directives refused PEG and four patients refused nasogastric intubation. Orotracheal intubation, tracheotomy, and advanced life support were refused by one patient each. All provided care was in accordance with the patient’s advanced directives. Of the patients who underwent PEG, two of them survived approximately three months, and one patient was submitted to a nasogastric tube less than a month before dying, thus suggesting that they were submitted to unnecessary invasive and painful procedures during their last weeks of life. Scarce evidence suggests that gastrostomy improves survival exists and, in some cases, its use for nutritional support is of little benefit and can be associated with increased mortality [9].

Patients with ALS often have multiple uncomfortable symptoms that severely impair quality of life, including pain, fasciculations, sialorrhea, pseudobulbar affect, spasticity, cramps, depression, anxiety, insomnia, and fatigue [1]. In our study population, the five most common symptoms were dysphagia, dyspnea, pain, anxiety, and sialorrhea. Dysphagia is reported within two years of disease onset by around 60% of patients with spinal-onset ALS and by all patients with bulbar-onset disease [10]. The second most frequent symptom was dyspnea. With disease progression, patients develop dyspnea at rest, poor cough, and respiratory tract infections; these problems are exacerbated by bulbar dysfunction [5]. Pain was the third most common complaint. When it comes to pain in ALS, it can be present in 15-85% of patients, depending on the duration of the disease, it is more commonly nociceptive than neuropathic [11]. When it comes to anxiety, it is a relevant and common component of the psychological constellation of symptoms that affects ALS patients and caregivers, in particular during the period surrounding the diagnosis, but also during disease progression [12]. Sialorrhea, which causes drooling and pooling of saliva within the oral cavity, is one of the most disturbing symptoms in patients with ALS and is commonly observed in patients with bulbar-onset disease and during the late stages of disease [10]. Although primary symptoms of ALS are associated with motor dysfunction, up to 40% of patients develop cognitive and/or behavioral impairment during the course of the disease [1]. However, this was not the case in this population, through the application of the CAM, it was determined that delirium was present in only one patient (5.00%).

When it comes to management, ALS symptoms can be treated with pharmacological and non-pharmacological interventions. In the early stages, management focuses on maximizing function, promoting independence, and treating symptoms [4]. Management of most ALS symptoms has not been rigorously evaluated and as a consequence, most recommendations for symptom management were decided by expert consensus and supported by treatment suggestions [2]. When it came to our study, we found that the most common drugs used were in line with the most frequent symptoms previously stated, as was to be expected. The five most used drugs were morphine, riluzole, butylscopolamine, bisacodyl, and midazolam. Morphine can be used and is effective not only for pain management but also dyspnea [10]. When it comes to riluzole, this drug is the only one to have shown a small survival benefit of approximately three months and has been approved for ALS treatment [5]. Butylscopolamine is an anticholinergic drug and is useful for respiratory secretion control, especially at the end of life [4]. Constipation can be a common disease symptom but also a drug side-effect, causing great discomfort, so the use of laxatives is frequent [10]. Bisacodyl was the most used laxative in this population. Midazolam is a very common drug in a palliative care setting, in this context it can be useful to treat such symptoms as anxiety, emotional lability, and spasticity but is also very important for palliative sedation during the last days of life when there are refractory symptoms [5].

Most patients with ALS die from respiratory failure. NIV is the preferred treatment for respiratory failure symptoms and can significantly improve quality of life [13]. However, in this study, we found that only 55% of patients (n=11) were submitted to NIV. This might be explained by the late involvement of palliative care, with almost a third of patients (30.00%, n=6) having their first consult with a palliative care physician on their last month of life, and of these patients, 83.33% (n=5) were on their last two weeks of life. By this stage, NIV might not be an appropriate treatment option due to the patient’s overall deterioration and closeness to death, where pharmacological intervention for symptom management was prioritized.

Although pathogenesis and disease course are heterogeneous, ALS is invariably and inexorably progressive, with survival varying greatly, ranging from several months to more than 10 years since symptom onset [14]. We found that 95.00% of our patients (n=19) died during this five-year period, with only one patient (5.00%) surviving, with a median life expectancy since diagnosis of 425 days (approximately 14 months). The median duration of palliative care was 137 days (approximately five months) and in some cases was only one day, which we have already established is not ideal and greatly limits a variety of interventions.

As for the patient’s preferred place of death, this is usually at home, but distressing symptoms, unanticipated crises, or increasing carer burden can make end-of-life care at home challenging [6]. We found that most patients (89.47%; n=17) died in a hospital setting, the majority of these (73.68%; n=14) in the palliative care unit, and three patients (15.79%) in other medical wards. Two patients (10.53%) were able to die at home. The place of death of ALS patients is indicative of different healthcare system structures and priorities, and so there are differences across countries. In some countries, we see a higher percentage of patients dying comfortably at home because there are support systems in place to allow this [15].

With this study we aimed to convey a faithful depiction of the ALS population that was referenced and/or followed by a palliative care unit; however, this study had some limitations, mostly due to sample size and symptom characterization. With a small study population of just 20 patients, the conclusions obtained are limited. In regards to symptom recording, this data might be undervalued, since it is influenced by the patient’s capacity and willingness to articulate their symptoms but also by the physician’s evaluation.

## Conclusions

Literature has shown that there are many advantages in early inclusion of palliative care in ALS management, achieving effective symptom control and greater quality of life, but also reducing caregiver burden. However, in this study, we found that referrals to palliative care were late and included mostly cases of advanced disease with uncontrolled symptoms. When it comes to ALS, data shows that death is inevitable, thus reinforcing the importance of a timely palliative care intervention, in order to reap the most benefits. With this in mind, there should be greater investment in training healthcare professionals to be more aware of the importance of palliative care and to promote early referrals, allowing for better management during disease progression and end of life.
